# Population genetics suggest that multiple invasion processes need to be addressed in the management plan of a plant disease vector

**DOI:** 10.1111/eva.12051

**Published:** 2013-02-13

**Authors:** Kylie L Anderson, Bradley C Congdon

**Affiliations:** School of Marine and Tropical Biology, James Cook UniversityCairns, Queensland, Australia

**Keywords:** colonization, invasion pathway, isolation by distance, long-distance dispersal, microsatellites, pest management, planthopper

## Abstract

The use of a multidisciplinary approach is becoming increasingly important when developing management strategies that mitigate the economic and biological costs associated with invasive pests. A framework of simulated dispersal is combined with life-history information and analyses of population genetic structure to investigate the invasion dynamics of a plant disease vector, the island sugarcane planthopper (*Eumetopina flavipes*), through an archipelago of significant Australian quarantine concern. Analysis of eight microsatellite loci from 648 individuals suggests that frequent, wind-assisted immigration from multiple sources in Papua New Guinea contributes significantly to repeated colonization of far northern islands. However, intermittent wind-assisted immigration better explains patterns of genetic diversity and structure in the southern islands and on the tip of mainland Australia. Significant population structuring associated with the presence of clusters of highly related individuals results from breeding *in-situ* following colonization, with little postestablishment movement. Results also suggest that less important secondary movements occur between islands; these appear to be human mediated and restricted by quarantine zones. Control of the planthopper may be very difficult on islands close to Papua New Guinea given the apparent propensity for multiple invasion, but may be achievable further south where local populations appear highly independent and isolated.

## Introduction

The environmental and economic costs of biological invasions and pest management are key issues for many countries (Pimentel et al. 1999). Understanding the various factors contributing to invasion success is important when seeking to implement effective control strategies for pest species. Foremost among factors influencing invasion success is the ability of an organism to disperse to new regions (Lockwood et al. 2007). If an invasive species is regularly transported along multiple pathways, the probability of its successful establishment is increased (Kolar and Lodge 2001). Likewise, certain life-history traits such as high reproductive capability are thought to favour establishment and spread (Williamson 1996).

Importantly, the dispersal and establishment history of an organism may be reflected in its spatial pattern of population genetic structure (Sakai et al. 2001; Excoffier et al. 2009). For example, multiple introductions via multiple dispersal pathways can increase genetic diversity, and as a consequence, there may be little genetic constraint to adaptation in a novel environment; thereby enhancing invasive capability (Kolbe et al. 2004). Not only do population genetic data provide valuable insight into the genetic consequences of invasion, they also provide information on movement pathways and invasion routes (Congdon et al. 1997; Suhr et al. 2010; Zepeda-Paulo et al. 2010) and overall invasion potential and pest status (Darling et al. 2008; Jiang et al. 2010). Taking a genetic approach to the management of invasive species and pests is gathering momentum; when coupled with ecological information on dispersal and/or colonization mechanisms and life-history characteristics, population genetics analyses provide a strong basis on which to formulate management strategies (Rollins et al. 2009).

**Figure 1:**
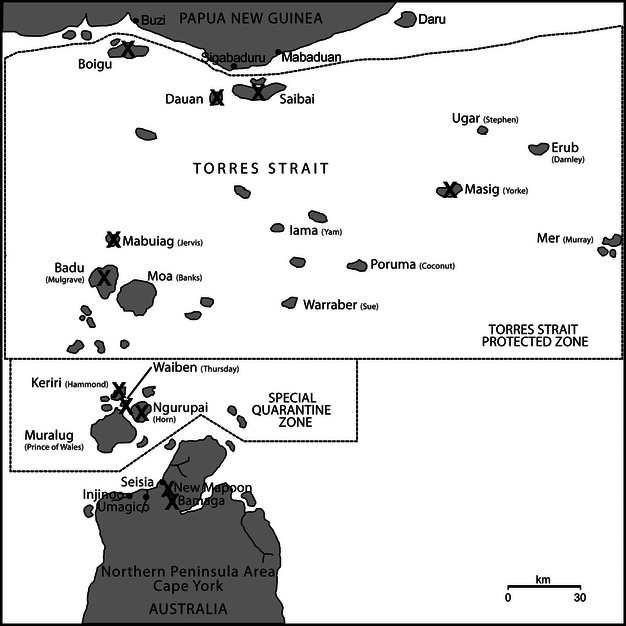
Southern coast of Papua New Guinea, Torres Strait islands and northern peninsula area, Australia showing potential Papua New Guinea source populations, *E. flavipes* TS/NPA sampling locations (marked with X) and quarantine zones (delimited by dashed lines).

The island sugarcane planthopper, *Eumetopina flavipes* Muir (Hemiptera; Delphacidae) is a significant invasive agricultural pest because it is the only known vector for Ramu stunt disease of sugarcane (Kuniata et al. 1994). In 1986, Ramu stunt significantly reduced commercial sugar yields in Papua New Guinea by over 60%, and the disease and vector remain of commercial significance today (Kuniata et al. 2001). Apparently, disease-free populations of *E. flavipes* occur in Australia, but are restricted to north-east Queensland in the Torres Strait islands (TS) and in the northern peninsula area (NPA) of Cape York (Fig. 1). The presence of *E. flavipes* in north-eastern Australia represents a significant quarantine threat to the commercial production of sugarcane in Australia, which occurs approximately 695 km south of the NPA (Sallam 2009).

*Eumetopina flavipes* and Ramu stunt disease are thought to be native to Papua New Guinea (PNG) (Kuniata et al. 1994; Wilson 2004). *E. flavipes* is widespread throughout PNG and resides only among the growing leaf rolls of four common host species of *Saccharum; S*. ‘hybrids’ grown mainly in commercial plantations at Ramu Agri-Industries, *S. officinarum* and *S. edule,* which are grown in residential gardens and *S. robustum* which grows wild and is highly abundant in suitable habitat throughout PNG (Paijmans 1976; Magarey et al. 2002). These four host types form a relatively continuous distribution across the landscape, probably promoting large and stable *E. flavipes* populations (Anderson et al. 2009).

A different situation exists in the TS/NPA, where *E. flavipes* occurs on the only two host plant types present, *S*. ‘hybrids’ and *S. officinarum*. Both are commonly cultivated in local gardens as either single plants, or in small patches that can contain multiple plants. Sugarcane may also grow untended and wild at some locations. Both long-term survey data (Gough and Petersen 1984; Chandler and Croft 1986; Allsopp 1991; Grimshaw 1997; Magarey 1997, 2003) and recent intensive sampling confirm that *E. flavipes'* presence in the TS/NPA is highly variable in both space and time, and that local extinctions and recolonization regularly occur (Anderson et al. 2009).

A number of potential dispersal pathways exist for *E. flavipes* in the Torres Strait. Simulation modelling suggests that southward wind-assisted migration of *E. flavipes* may occur from PNG into the TS/NPA during the summer monsoon season, where the resulting number of immigrants per island is a function of wind direction and distance from PNG (Anderson et al. 2010). However, there was no significant linear relationship between predicted immigration and observed patterns of infestation throughout the TS/NPA (Anderson et al. 2010). There were a small number of locations where wind-assisted immigration appeared to be a good predictor of observed infestation, but *E. flavipes* was absent at a number of other locations despite immigration being predicted (Anderson et al. 2010). A number of postcolonization factors could account for the discrepancy. For some species, these may include natural enemies and competitors, but these appear unlikely to impact on *E. flavipes'* distribution and abundance in the TS/NPA. Regular field surveys revealed that very few natural enemies were noted and there are no other *Eumetopina* species present that could compete for resources, as occurs in PNG. Anderson et al. (2009) note that host plant availability varies in the TS/NPA and is likely to affect the distribution and abundance of *E*. *flavipes*. However, the presence of alternate and/or additional dispersal pathways, such as the human-mediated movement of infested sugarcane, could easily facilitate *E. flavipes* dispersal among island and mainland communities, and contribute to the establishment of new populations.

Should anthropogenic movement of infested sugarcane occur, then *E. flavipes* movement should be restricted by the special quarantine zones that occur between PNG and mainland Australia. These zones have been established in a bid to halt the movement of pests and diseases that could damage Australia's animal and plant industries (Australian Government Department of Foreign Affairs and Trade 1985). Movement of ‘declared’ items is permitted within PNG, the Torres Strait Protected Zone, the Special Quarantine Zone and mainland Australia, but not between zones (Fig. 1). Sugarcane is a declared item, so effectively, there should be no anthropogenic movement of sugarcane between each quarantine zone.

In this study, we use microsatellite genetic markers to evaluate both large- and small-scale population genetic differentiation and connectivity within and among island and mainland populations of *E. flavipes* throughout the TS/NPA. On the basis of previous simulation modelling (Anderson et al. 2010), we hypothesize that *E. flavipes'* primary method of transport into the TS/NPA is long-distance, wind-assisted dispersal from PNG. If so, and in keeping with predicted patterns for long-distance dispersal, populations across the TS/NPA should show a decrease in genetic diversity and association with distance from PNG source populations, associated with fewer immigrants reaching peripheral sites (Austerlitz et al. 1997; Gillespie et al. 2012). Thus, our expectation is that data will not conform to either of the principal theoretical models (stepping-stone or island). Exceptions may suggest that alternate dispersal pathways are operating, such as human-mediated movement of infested sugarcane between islands. Therefore, we also test specifically for population genetic structuring within and among quarantine zones. Given what is generally known about planthopper colonization and establishment (Kuno 1979), we suspect that population growth following immigration will result primarily from matings among a limited numbers of colonists and subsequently their offspring, and we expect that this effect will be particularly strong at locations distant from putative PNG source populations. Thus, we test for family-associated genetic structuring within and among TS/NPA populations.

Eradication of *E. flavipes* from mainland Australia was suggested as far back as 1989 (Allsopp 1989), but no action has been taken. By combining patterns of genetic structure and connectivity with hypothesized models of dispersal, we are able to discuss findings of this study from an ‘island-specific’ approach to the management of this high-risk pest species in the TS/NPA, as well as demonstrate the benefits of such an approach in a broader invasive species-management context.

## Materials and methods

In 2006 and 2008, *E. flavipes* surveys were conducted throughout the TS and NPA. *Eumetopina flavipes* were collected for genetic analyses from *S. officinarum* and *S*. ‘hybrids’ grown at nine TS and two NPA communities; these communities were termed locations (Table 1). Due to the haphazard nature of island sugarcane cultivation, collections were made from what we defined as a host ‘patch’, or a stand of isolated sugarcane, which in some cases contained a single plant, but in other cases contained numerous plants grown in such close proximity to each other that the stalks and leaves were intertwined and impossible to separate. The vast majority of patches occurred in residents gardens. *E. flavipes* sampled from a single patch were defined as a population (Table 1). Ideally, 25 individuals were collected via aspiration from five randomly selected stalks per patch, and transferred immediately to 100% ethanol. If less than 25 individuals were available on the five focal stalks, then where possible, further samples were randomly collected from additional stalks within the same patch. Representative adult subsamples were submitted to Delphacidae taxonomist, G. A. Bellis, Darwin, Australia to confirm identification. Voucher specimens from four locations, being Bamaga, New Mapoon, Badu and Saibai, were lodged with the Queensland Museum, Brisbane, Australia.

**Table 1:** *E. flavipes* collection details (*n* = sample size)

Quarantine zone	Location	Population	*n*	Year	GPS coordinates
NPA	Bamaga	1	26	2006	10°53′37.97″S
					142°23′20.82″E
		2	27	2006	10°53′22.48″S
					142°23′24.92″E
		3	25	2008	10°53′37.97″S
					142°23′20.82″E
		4	25	2008	10°53′22.48″S
					142°23′24.92″E
	New Mapoon	1	25	2006	10°52′10.56″S
					142°23′0.35″E
		2	17	2006	10°52′17.37″S
					142°23′8.04″E
SQZ	Keriri	1	22	2008	10°33′12.83″S
					142°13′2.11″E
	Waiben	1	25	2006	10°34′55.69″S
					142°13′19.49″E
	Ngurupai	1	25	2006	10°35′43.82″S
					142°14′57.39″E
		2	25	2006	10°35′34.85″S
					142°14′53.96″E
		3	21	2008	10°35′38.99″S
					142°14′56.96″E
TSPZ	Masig	1	20	2006	9°45′0.40″S
					143°24′52.21″E
		2	15	2006	9°45′1.71″S
					143°24′46.76″E
		3	9	2006	9°45′5.83″S
					143°24′38.94″E
	Mabuiag	1	14	2006	9°57′10.26″S
					142°11′32.01″E
		2	13	2008	9°57′25.00″S
					142°11′13.73″E
		3	9	2008	9°57′25.43″S
					142°11′13.23″E
	Badu	1	25	2006	10° 9′1.03″S
					142°10′12.33″E
		2	13	2006	10° 9′0.17″S
					142°10′13.14″E
		3	25	2008	10° 9′20.35″S
					142°10′6.00″E
	Dauan	1	25	2006	9°25′8.19″S
					142°32′29.68″E
		2	16	2008	9°25′7.01″S
					142°31′46.87″E
		3	25	2008	9°25′8.19″S
					142°32′29.68″E
	Saibai	1	25	2006	9°22′54.07″S
					142°36′42.39″E
		2	25	2006	9°22′37.29″S
					142°37′32.80″E
		3	25	2008	9°22′52.12″S
					142°36′40.99″E
		4	25	2008	9°22′34.08″S
					142°37′25.68″E
	Boigu	1	25	2006	9°13′48.63″S
					142°13′8.68″E
		2	25	2006	9°13′50.22″S
					142°13′11.74″E
		3	11	2008	9°13′51.81″S
					142°13′13.38″E
		4	15	2008	9°13′48.93″S
					142°13′16.05″E

Due to low sample numbers collected in either year, populations sampled in both 2006 and 2008 were used in the analyses described below. Each population was assigned a unique identifier and analysed independently so that temporal variation was identifiable (Table 1).

### DNA methods and microsatellite characteristics

Microsatellites were developed specifically for this study (Table 2). The novel microsatellite and primer sequences were submitted to GenBank (Locus name and GenBank accession number respectively: *1-TER-327* JN565018; *2-TER-427* JN565019; *3-TER-527* JN565020; *4-TER-627* JN565021; *5-TER-727* JN565022; *6-TER-827* JN565023; *7-TER-1027* 565024; *8-TER-10* JN565025). Whole insects were sent to the Australian Genome Research Facility Limited for their standard DNA extraction, PCR amplification and microsatellite genotyping at eight polymorphic loci. For PCR, initial denaturing was at 94°C for 5 min, 35 amplification cycles of 94°C for 30 s, annealing temperature (Table 2) for 45 s and 1 min of extension at 72°C, with a final extension at 72°C for 3 min, with samples held at 4°C. Applied Biosystems (Victoria, Australia) 3730 DNA Analyser platform with a GeneScan -500LIZ size standard was used for electrophoresis. Standard GeneMapper 4.1 software (Applied Biosystems) was used for scoring alleles.

**Table 2:** Annealing temperature (*T*_a_), number of alleles (*A*), inbreeding coefficient (*F*_IS_ - asterisk (*) indicates significance at *P* = 0.05), per loci for eight pairs of novel microsatellite primers

Loci	Sequence (5′–3′)	Repeat	Clonedallele size	*T*_a_ (^o^C)	*A*	*F*_IS_
*8-TER-10*	F TTTGCTGTCAACTCCCATTG	(AC)_3_ (AC)_24_	189	55	25	−0.0252
	R GATGAGAGATGACAAGA					
*1-TER-327*	F TGAGGCGTGGCTGCTAGT	(AC)_16_	164	52	23	0.1451*
	R CATTTCCATTAGTAATTTTCCCTCA					
*2-TER-427*	F TCATTTCAGCAAATTGTGAGC	(AC)_11_ (AC)_9_	135	52	15	−0.0189
	R CCCTATGATCACTTAGCAACCA					
*3-TER-527*	F GGAATACTGGGTGTGAGTTGC	(CA)_7_ (CA)_7_	170	55	45	−0.0415
	R AATGAGGCCGACTTGTATGC					
*4-TER-627*	F GCTCACGTTCAAGCTTCCTC	(CA)_10_	195	55	24	−0.0539
	R GAGGGGAGAGGGAGTGAGAG					
*5-TER-727*	F TGCATGGGTAATGAAGTGGA	(CA)_6_ (CA)_7_ (CA)_11_	202	52	28	0.0057
	R GTAATGGACGGGCTACAGGA					
*6-TER-827*	F GCCTGGCACTCACATACACA	(CA)_16_	122	52	17	0.4747*
	R TCACTAGCTTGCAGTTTGCTG					
*7-TER-1027*	F TTCTGGCATACTGGGTGTGA	(CA)_3_ (CA)_6_	153	52	9	−0.1334
	R CCGGCAGATAGGAGTTTGAG					

The presence of null alleles, scoring error due to stuttering and large allele dropout were tested using Microchecker 2.2.3 (Van Oosterhout et al. 2004). Cervus 3.0.3 (Kalinowski et al. 2007) was used to estimate the frequency of null alleles per locus. Linkage disequilibrium was analysed using the likelihood ratio test, with 10 000 permutations in Arlequin 3.5.1.2 (Excoffier et al. 2005).

Population genetic characterization was done by calculating the expected (*H*_E_) and observed heterozygosity (*H*_O_) in Arlequin. Locus-by-locus departure from Hardy–Weinberg equilibrium was tested by determining significance of the inbreeding coefficient *F*_IS_ (heterozygosity deficit), with 10 000 permutations in Arlequin. All multiple comparison *P* values were corrected for false discovery rate (Benjamini and Hochberg 1995). The allele size permutation test described in Hardy et al. (2003) was used to ensure *F*-statistics were an appropriate measure of population genetic differentiation for our data. The multilocus *R*_ST_ value was not significantly higher than the mean *pR*_ST_ (*P* = 0.564), therefore, *F*_ST_ was suitable.

Isolation by distance was examined in the first instance to estimate the magnitude of inter-population gene flow throughout the TS/NPA. A Mantel test was conducted with pairwise Slatkin's linearized *F*_ST_ [*F*_ST_/(1−*F*_ST_)] and the natural log of pairwise geographical distances between locations in the TS/NPA (Rousset 1997). Significance was assessed with 9999 permutations in Arlequin. To calculate the geographical distances, latitude and longitude coordinates recorded in the TS/NPA on a Garmin GPS 60 device were uploaded to Google Earth 5.1.3533.1731 (Google Inc. 2009). Pairwise distances between TS/NPA sampling locations were calculated using the Google Earth ruler tool at ‘eye view’ 1 km above the ground for consistency.

To examine the applicability of the wind-immigration hypothesis, we used linear regression analyses to test for an effect of geographical distance from known *E. flavipes* infestations along the southern coast of PNG. *E. flavipes* has been recorded on many occasions as abundant on sugarcane grown in the southern coastal PNG villages of Sigabaduru, Mabaduan, Daru and Buzi (Waterhouse et al. 1995; Grimshaw 1999; Magarey et al. 2002) (Fig. 1). We hypothesized that these populations could easily act as point immigrant sources for wind-assisted migration into the TS/NPA. GPS coordinates for each of these four potential source populations along with GPS coordinates for each sampled TS/NPA infestation were uploaded to Google Earth. Distances between each TS/NPA population and the closest PNG village infestation were measured using the Google Earth ruler tool, as described previously, and used as the predictor variable in linear regression analyses.

Allelic richness, observed heterozygosity and population-specific *F*_ST_ were used as dependent variables in the above regressions. Allelic richness and heterozygosity are important measures of population genetic diversity (Petit et al. 1998), while population-specific *F*_ST_ is particularly useful for estimating the genetic uniqueness of individual populations within a group of populations, especially when used in conjunction with allelic richness (Gaggiotti and Foll 2010). Allelic richness was standardized using rarefaction in the program HP-RARE v June-6-2006 (Kalinowski 2005). Mean population *F*_ST_ was calculated in the program Geste (Foll and Gaggiotti 2006).

Overall genetic differentiation throughout the TS/NPA was assessed with *F*_ST_ (Wright 1965) using analysis of molecular variance (AMOVA). Spatial hierarchical amova was used to test whether significant genetic differentiation occurred between quarantine zones within the TS/NPA. Populations were grouped for the analysis as follows: (i) mainland Australia (NPA) (Bamaga and New Mapoon), (ii) Special Quarantine Zone (Keriri, Waiben and Ngurupai) and (iii) Torres Strait Protected Zone (Masig, Mabuiag, Badu, Dauan, Saibai and Boigu). Significance of *P* was assessed with 10 000 permutations in Arlequin.

In addition to classic *F*_ST_ analyses, the Bayesian clustering method implemented in Structure v 2.2.3 (Falush et al. 2003) was used to test for evidence of population genetic structuring, assign individuals to populations and to identify admixed individuals (Falush et al. 2003). As the number of genetic clusters (*K*) in our data was unknown, Structure was used to assign individuals into the most likely *K*. We evaluated results for *K* = 1 to *K* = 33, being no genetic structure at all (*K* = 1) to every sampled population being genetically distinct (*K* = 31). Estimates of *K* were based on 10 iterations, each with a burn in of 50 000, and Markov Chain Monte Carlo (MCMC) lengths of 100 000 using the admixture model and correlated allele frequencies. The optimal value of *K* was based on both log probabilities [Pr (X|K)] and Δ*K* (Evanno et al. 2005). Summary outputs were viewed using Structure harvester v 0.6.1 (Earl 2011). Individual assignment to a particular cluster was based on the largest average proportion of their genotype assigned to a cluster over the 10 iterations.

As we hypothesize that there will be related individuals within populations, a maximum-likelihood model, Colony v 2.0.1.1 (Jones and Wang 2010), was used to analyse specifically for genealogical relationships within and between populations, by reconstructing sibling relationships (sib-ships). Individuals (*n* = 648, 7 loci; Locus *6-TER-827* was not used in the Colony analysis due to the presence of null alleles, see results) were pooled, and an allelic dropout rate of 0.1%, and 1.5% for other errors were assumed. Female monogamy and male polygamy were selected because it appears unlikely that female planthoppers mate repeatedly, whereas multiple mating by males is common (Claridge and Vrijer 1994). Three long and three medium runs were conducted, each with different random seed numbers. Results were tested for convergence by plotting the change in Log-likelihood as a function of the number of iterations (Jones and Wang 2010), and only inferred sib-ships with a probability over 0.9 were plotted.

## Results

A total of 648 individuals from 31 populations were sampled from locations throughout the TS/NPA (Table 1). All eight loci were polymorphic, with a total of 186 alleles. No scoring errors or allele drop out were detected. *F*_IS_ values at *1-TER-327* and *6-TER-827* were significant (Table 2). However, the *F*_IS_ value at *1-TER-327* was closer to the range of *F*_IS_ values at other loci, except for *6-TER-827* (see below). Running analyses with and without *1-TER-327* did not significantly change any outcomes, so this locus was included in all analyses.

The *F*_IS_ value at *6-TER-827* was an order of magnitude larger than that of other loci (Table 2). Furthermore, 77% of populations showed significant deviation from Hardy–Weinberg equilibrium at this locus, and Microchecker indicated that it was probably due to the presence of a null allele. Cervus estimated the frequency of null alleles at *6-TER-827* to be 0.52. Consequently, this locus was excluded from all further analyses. Significant deviation from Hardy–Weinberg equilibrium occurred at five of seven loci at Keriri 1, five of seven loci at Mabuiag 1 and six of seven loci at Dauan 2. However, there was no significant global deficit of heterozygotes in these populations (multilocus population *F*_IS_ values not significantly different from zero). Isolation by distance calculations were conducted with and without these three populations. In no instance did their inclusion alter result significance, so results presented are from analyses containing all populations.

Significant linkage disequilibrium occurred in most populations even after correction. This result was not unexpected, given that populations in this study may have been influenced by evolutionary processes such as founder effects and inbreeding following introduction, which are known to cause linkage disequilibrium (Slatkin 2008). Importantly, no loci were consistently linked across multiple populations, so it was assumed loci assorted independently for statistical testing. A summary of locus variation can be found in Anderson (2011).

**Figure 2:**
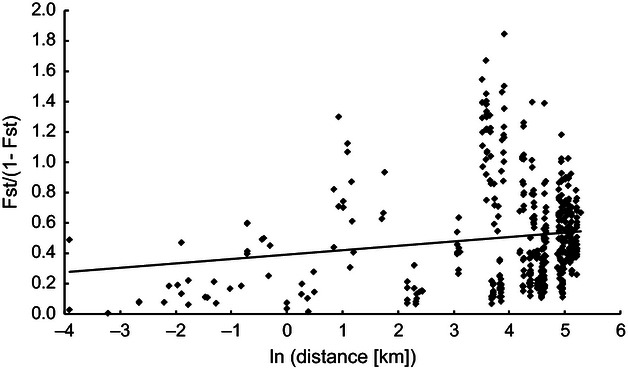
Relationships between the natural log of geographical distance and Slatkin's linear genetic distance for *E. flavipes* on the Torres Strait islands and northern peninsula area, Australia.

Isolation by distance analysis revealed a significant positive correlation between Slatkin's linearized *F*_ST_ and the natural log of geographical distance (Mantel *r* = 0.15, *P* = 0.0003) (Fig. 2). Despite the significance of this relationship, the Mantel *r* value of 0.15 suggests that geographical distance between populations is a relatively poor predictor of genetic differentiation. High levels of genetic variability were consistently observed between pairwise Slatkin's linearized *F*_ST_ values regardless of distance between populations, suggesting that alternate factors are probably contributing to the observed genetic structuring.

**Figure 3:**
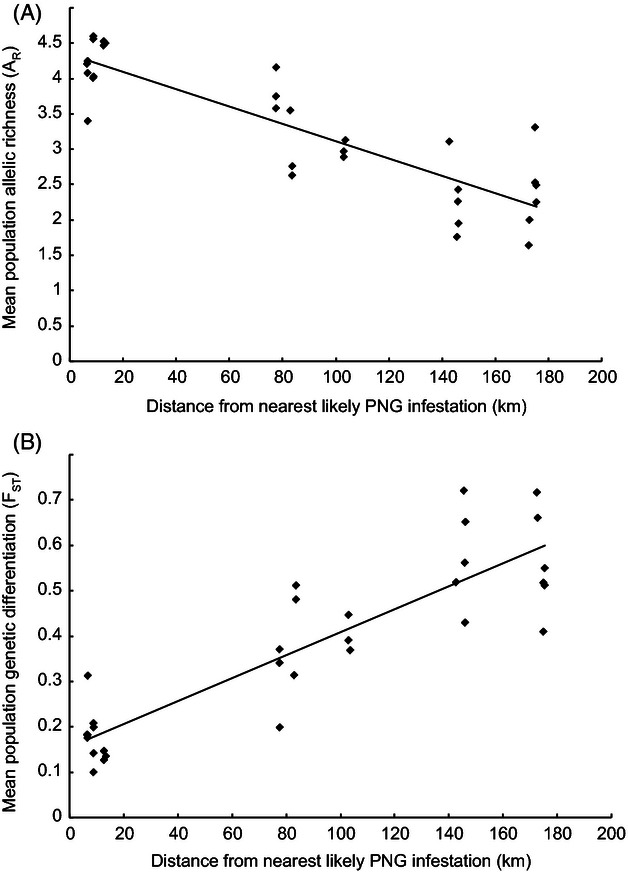
Relationship between distance from the nearest likely Papua New Guinea source population and (A) mean population allelic richness (A_R_) and (B) and mean population genetic differentiation (*F*_ST_) in the Torres Strait and northern peninsula area, Australia.

Mean population allelic richness was significantly negatively related to distance from PNG, with distance explaining 75% of the variation in allelic-richness differences between sites (Adj *R*^2^ = 0.75, *F*_1, 29_ = 91.57, *P* < 0.001; Fig. 3A). Similarly, observed heterozygosity was significantly negatively related to distance from PNG, with distance explaining 32% of the variation in observed heterozygosity (Adj *R*^2^ = 0.32, *F*_1, 29_ = 13.81, *P* = 0.001). Thus, population genetic diversity decreases with increasing distance from PNG. Conversely, mean population *F*_ST_ significantly increased with distance from PNG, which explains 77% of the variation in *F*_ST_ (Adj *R*^2^ = 0.77, *F*_1, 29_ = 101.38, *P* < 0.001; Fig. 3B). Population genetic structuring thus increases with distance from PNG.

A global *F*_ST_ of 0.32 (*P* < 0.001) suggests that significant population genetic differentiation occurs throughout the TS/NPA. Of the total variation, 68% occurred within populations, with the remaining 32% variation among populations also significant. After correction for false discovery rate, 463 of the 465 population pairwise *F*_ST_ comparisons were significant. Populations sampled in both years from the same host plant patch were significantly genetically different between years of sampling (Bamaga 1 and 3 *P* = 0.043; Bamaga 2 and 4 *P* < 0.001; Dauan 1 and 3 *P* = 0.01). Only Badu 1 and 2 (both sampled 2006) and Saibai 3 and 4 (both sampled 2008) were not significantly different from each other (pairwise *F*_ST_ = 0.004, *P* = 0.29; pair-wise *F*_ST_ = 0.014, *P* = 0.07, respectively). Results of the hierarchical amova indicated that significant regional structuring also occurs, where 16.6% of the overall variation was attributed to quarantine zone. However, only 20.06% variation occurs among populations within quarantine zones, which is low when compared with the 63.34% of variation that occurs within populations (*F*_ST_ = 0.37; *F*_SC_ = 0.24, *F*_CT_ = 0.17; *P* < 0.001 for each level of variation). So although significant, grouping populations by quarantine zone only weakly explains population genetic structuring in the TS/NPA. The majority of genetic differentiation is explained at the individual population level.

**Figure 4:**
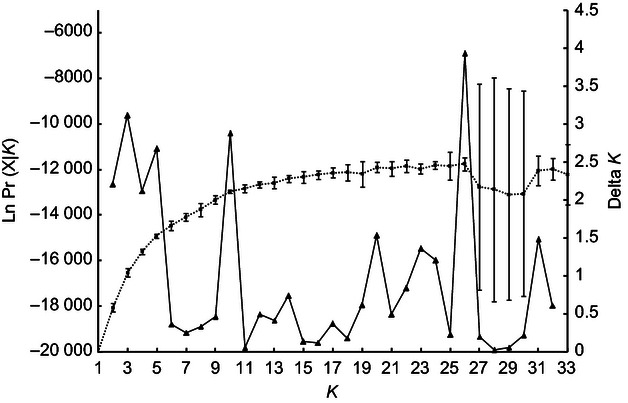
Results from Structure analysis for *K* = 1–33 populations sampled throughout Torres Strait and northern peninsula area, Australia, with average log likelihood LnPr (X|*K*) (± SD) on the primary axis, Δ*K* values on the secondary axis.

**Figure 5:**
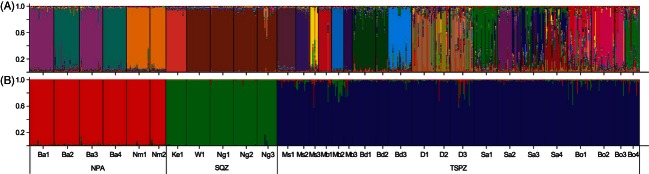
Structure *Q* plots (A) *K* = 26; (B) *K* = 3. Populations: Bamaga (Ba), New Mapoon (Nm), Keriri (Ke), Ngurupai (Ng), Masig (Ms), Mabuiag (Mb), Badu (Bd), Dauan (D), Saibai (Sa) and Boigu (Bo); grouped by northern peninsula area (NPA), Special Quarantine Zone (SQZ), Torres Strait Protected Zone (TSPZ). Sampled individuals are represented by a vertical bar showing the degree of admixture.

Structure analyses indicated the highest average log likelihood occurred at *K* = 26 (−11745.70) (Fig. 4). Using the Evanno method (Evanno et al. 2005), the Δ*K* statistic peaked at *K* = 26 (3.93) (Fig. 4). Examination of the *α* plots revealed very little variation, suggesting that the burn-in and run-times were sufficient for convergence (Falush et al. 2003). Structure results support those of the amova, suggesting that strong population genetic structuring occurs in the TS/NPA. Within our data, strongest support exists for 26 distinct genetic clusters, and close examination of the Structure *Q* plot revealed two general patterns (Fig. 5A). First, the majority of individuals were strongly assigned to the population from which they were sampled, and this effect is strongest on the NPA and for locations in the southern TS, especially those within the Special Quarantine Zone. Interestingly, all the individuals sampled from Waiben 1 and all three Ngurupai populations formed a single cluster (Fig. 5A). Second, as distance to PNG decreases, individuals are much less strongly assigned to the population from which they were sampled because individual levels of admixture are increasing (Fig. 5A). Δ*K* also suggests that levels of substructuring occur, with peaks at *K* = 3 (3.11) and *K* = 10 (2.88) (Fig 4). *K* = 3 was further examined to determine if the clusters contained populations grouped according to quarantine zone, which they did (Fig. 5B). Evanno et al. (2005) note that Structure is able to detect complex hierarchical levels of genetic structure, but Falush et al. (2003) warn that while the ultimate *K* should capture most of that structure, there should be a sound biological reason to explain it. For our data, the strongest support exists for *K* = 26 and *K* = 3, so we conclude that despite there being some support for *K* = 10, it may represent yet a further layer of genetic substructure, but for which there is no clear biological cause.

**Figure 6:**
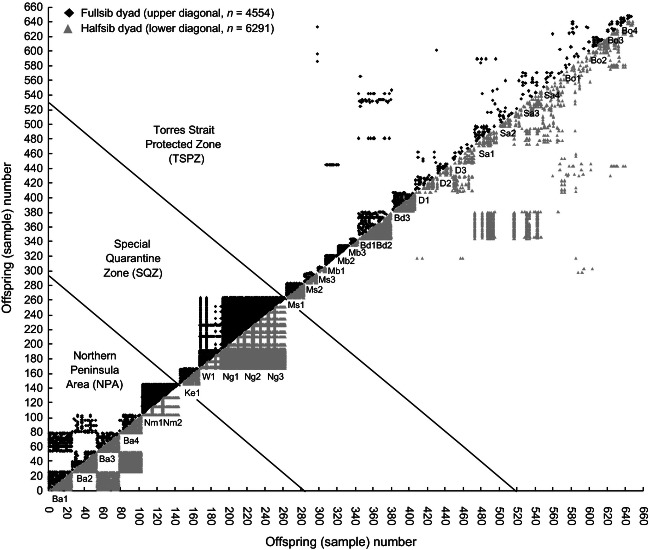
Plot of best (maximum likelihood) sib-ship assignment (*n* = 648) in the Torres Strait and northern peninsula area, Australia. The lower half of the matrix shows pairwise half-sib relationships, while the top half shows pairwise full-sib relationships. Grouped by populations: Bamaga (Ba), New Mapoon (Nm), Keriri (Ke), Ngurupai (Ng), Masig (Ms), Mabuiag (Mb), Badu (Bd), Dauan (D), Saibai (Sa), Boigu (Bo) and northern peninsula area (NPA), Special Quarantine Zone (SQZ), Torres Strait Protected Zone (TSPZ).

The Colony analysis suggests the presence of significant family structure throughout the TS/NPA; a total of 10 845 dyads (4554 full-sib and 6291 half-sib) occurred with over 0.9 probability. Plots of the change in Log-likelihood values as a function of the number of iterations from each of the replicate runs were consistent, indicating that the annealing procedure produced convergence and was powerful (Jones and Wang 2010). Individuals were assigned to their correct sampling location close to the Australian mainland, suggesting a high degree of reliability in the overall assignments (Fig. 6).

The sib-ship pattern surrounding Boigu, Saibai and Dauan, all adjacent to the coast of PNG, appears ‘scattered’ as a result of sib-ships between multiple populations and locations (Fig. 6). Although not the dominant sib-ship pattern, this scatter effect extends to Badu. In contrast, the pattern of sib-ships appears ‘linear’ for the remaining islands mostly as a result of sib-ships occurring between individuals within a single population. To a lesser extent, sib-ships also occur either between populations within a single location (e.g. between New Mapoon 1–2), or between populations across two locations (e.g. across Waiben and Ngurupai).

Colony suggests that two individuals sampled from Waiben 1 were full-sibs to the majority of individuals at Ngurupai 3 (patch not present in 2006, present and positive for *E. flavipes* in 2008), and many individuals sampled at the two locations were half-sibs. Waiben and Ngurupai islands are geographically ‘next door’. When the owner of the sugarcane plants at Ngurupai 3 was interviewed, she stated that she had obtained her plants from Waiben 1. Similar linear sib-ships occur across New Mapoon 1–2, Waiben 1 and Ngurupai 1–2, as well as between Ngurupai 1, 2 and 3. Further examples of such directional across-location sib-ships are evident between Badu 1–2 and Saibai 1, 2 and 3, where a number of individuals from Saibai are related at both full- and half-sib level to the majority of individuals at Badu 1–2.

## Discussion

*Eumetopina flavipes* populations on islands close to PNG exhibit significantly higher genetic diversity, higher levels of admixture and lower population-specific genetic structuring than populations closer to mainland Australia. These results combined with the apparently random assignment of individuals from islands close to PNG to clusters by Structure, and the dominant ‘scattered’ pattern of interpopulation sib-ship relationships observed in this region supports the founding of these populations by either multiple independent introductions from a number of genetically diverse source populations (Allendorf and Lundquist 2003; Kolbe et al. 2004; Chu et al. 2011), or a single large highly diverse source (Colautti et al. 2005) in PNG. *Eumetopina flavipes* also appears to conform to a general expectation of random distribution of founder populations, which appears the norm for a number of other planthopper species (Perfect and Cook 1994).

Theoretically, a unidirectional stepping-stone model of progressive range expansion away from source populations in PNG should produce a decrease in genetic diversity along the expansion axis and clear associations among adjacent populations (Austerlitz et al. 1997; Excoffier et al. 2009). However, pairwise comparisons and Structure clustering of our data suggest this is not the case, with the majority of individuals clustering into genetically distinct, independent aggregations corresponding to the population from which they were sampled. In addition, sib-ships occur between islands that are not always adjacent, so a consistent north-to-south, progressive ‘island-hop’ mode of dispersal is not supported. We suggest that increasingly rare, long distance founding events by relatively fewer individuals are responsible for this pattern of interisland variation. This is in keeping with wind trajectory modelling for *E. flavipes* (Anderson et al. 2010), suggesting that long-distance, wind-assisted dispersal from PNG, rather than interisland movement, is primarily responsible for *E. flavipes* immigration into the TS/NPA.

If the dispersal mode and distance can be predicted for a particular taxa, then so too can the resulting spatial patterns of connectivity and divergence upon arrival (Gillespie et al. 2012). Results from a number of studies that compare prevailing wind direction with population genetic data in planthoppers and other arthropods support our findings that *E. flavipes* engages in a seasonal migration from PNG. Analysis of brown planthopper *Nilaparvata lugens* mtDNA showed higher haplotype diversity in northern populations; a result consistent with a seasonal, northward migration from south-eastern China to Korea as predicted by weather patterns (Mun et al. 1999). A study on white-backed planthopper *Sogatella furcifera* found significant genetic differentiation between sampled regions, and patterns of population clustering suggested that northern *S. furcifera* migrated from a number of southern source locations (Liu et al. 2010). In Australia, levels of admixture across northern and southern *Bemisia tabaci* populations reflect prevailing wind trajectories at a time of year when the whiteflies are most active (De Barro 2005). While highly relevant for sap-feeding pests, patterns of population genetic structuring have been shown to support similar predictions regarding the origin of individuals, dispersal pathways and spread for a range of other taxa, such as reptiles (Kolbe et al. 2004), mammals (Cote et al. 2012) and birds (Rollins et al. 2009).

A general ‘colonisation syndrome’ has previously been described for migrating planthoppers, where low initial colonization densities are followed by little local-scale movement and rapid *in-situ* population growth (Kuno 1979). For example, limited dispersal of nymphs and adults within host patches following colonization has been shown to result in strong aggregations for the planthopper *Delphacodes scolochloa* (Cronin 2009), and other planthopper species (Perfect and Cook 1994). Our results suggest that *E. flavipes* conforms to this colonization syndrome, with population growth being predominantly kin-structured which, along with apparent relative isolation following colonization, serves to enhance founder effects promoted by wind immigration to ensure that strong genetic differentiation between populations persists over time.

Interestingly, previous research noted that different colour forms occurred in the Torres Strait, where dark and light colour variants were collected from Saibai (Allsopp 1991). Visual inspection of samples collected for this study by the primary author (K. L. Anderson, unpublished data) revealed that very light colour forms generally occurred on the NPA, while the darker forms occurred closer to PNG, and samples collected from PNG and Indonesia appeared much darker again. We speculate that such colour variation is a result of genetic divergence due to isolation, and this effect appears to be strongest among NPA populations.

Results from the sib-ship and Structure analysis suggest that secondary movement of *E. flavipes* occurs between and within locations via an alternate dispersal pathway. Sib-ships occur between individuals from Waiben and all three Ngurupai populations, and Structure clusters the same four populations together. Anecdotally, the sugarcane plants at Ngurupai 3, which were not present in 2006, were sourced from Waiben. Thus, the linear pattern of sib-ships across these two locations most probably represents human-mediated, directional movement of infested sugarcane. The movement of live individuals could occur, as adults and nymphs can survive at least six days on cut sugarcane stalks (Anderson et al. 2007), and viable eggs present in the leaf vein can hatch after stalk transplantation (K. L. Anderson, unpublished data). In addition, populations at New Mapoon 1 and 2 are directly connected via related individuals, as are populations on Badu 1 and 2 to Saibai 1 through 4; these relationships also likely represent human-mediated movements. Significant hierarchical amova and Structure clustering at *K* = 3 further support our hypothesis that secondary movements occur and that they are in fact restricted by quarantine zone.

As for many phytophagous animals, planthoppers are entirely dependent on the presence of suitable host plants (Denno and Perfect 1994). Anderson et al. (2009) suggested that the availability of *E. flavipes* host plants in the TS/NPA is severely impacted by local cultivation practices in the following way. Standard practice throughout the islands appears to involve the annual harvesting and removal of all sugarcane plants, although variability between locations in the actual timing of harvest was noted. Leaf material (upon which *E. flavipes* resides) is removed from the stalk, dried in the sun and then burned, while the stalks are cut into smaller pieces and replanted. Levels of postplanting care varied, and appeared to determine whether the stalk would successfully grow. Long-term detection records indicate that *E. flavipes* has been present in the TS/NPA for at least 28 years, despite such cultivation practices. Intensive sampling during this study revealed that within a 2-year period local extinction/recolonization events occur and may be driven by cultivation practices as described above that remove entire host plants, causing the distribution and abundance of *E. flavipes* populations to change in both space and time (see Anderson et al. 2009). Individual populations may thus be transient because of variation in host plant availability, but long-term regional persistence still occurs.

Results suggest that long-term persistence is achievable because at the broadest scale, recolonization is dominated by wind-assisted, long-distance immigration from PNG, which may occur annually (Anderson et al. 2010). Human-mediated local movements may also occur, but appear of relatively less importance. Multiple introductions via the use of multiple dispersal pathways likely enhance an invasive species' ability to occupy new areas and/or recolonize invaded areas because of the increased propagule pressure (Grevstad 1999; Simberloff 2009). This may be especially true for populations on islands adjacent to PNG, but in addition, these populations could be more robust to environmental selection pressures due to their high genetic variability, and potential for subsequent rapid evolution and adaptation (Dlugosch and Parker 2008).

Conversely, the relatively low levels of genetic variation exhibited by the southern populations might imply limited persistence over time, which is not the case. Low genetic diversity within recently invaded populations, as a result of founder effects, bottlenecks and genetic drift, does not always appear to be a barrier to successful invasion and subsequent population growth (Darling et al. 2008; Bai et al. 2012). This may be especially true for populations when accompanied by behaviour that enhances establishment and ongoing success, such as kin-structured population growth (Ingvarsson and Giles 1999).

In other systems, eliminating the dispersal pathway and/or a focus on reducing the size of the source population have been suggested for pest management (Russell et al. 2009; Zalewski et al. 2010); but neither of these are viable for *E. flavipes* in the TS/NPA. Previous research suggests that cultivation practices that remove host plants (e.g. annual removal, burning and replanting of stalks as discussed previously) could significantly reduce *E. flavipes* infestation, and if publicly encouraged could achieve local or even regional eradication (Anderson et al. 2009). However, results from this study suggest that only a temporary reduction in population size may be achievable with such a strategy, and that permanent eradication of *E. flavipes* is unlikely, especially on islands close to PNG given the apparent propensity for successful invasion.

Our results suggest that the type of management employed for *E. flavipes* should be location specific. This is implied because populations in the northern TS that exhibit higher levels of genetic diversity will be more difficult to manage than those on the NPA, principally because of much higher levels of propagule pressure (Lockwood et al. 2007). We suggest that in the first instance, a one-off, TS/NPA-wide effort focused entirely on tip pruning, in effect removing *E. flavipes* favoured host material, and then followed by annual monitoring and further location-specific tip pruning if recolonization is detected, may achieve longer lasting control. Such a strategy may achieve permanent eradication in the southern TS given the apparent lower invasion pressure and reproductive isolation. There is some evidence that quarantine zones restrict gene flow throughout the region, but ultimately, anthropogenic movement cannot be prevented. However, a continuing tip-pruning management strategy would reduce the likelihood of an infested stalk being moved.

Invasions are often complex; results from this research show that *E. flavipes* is no exception. This study thus demonstrates how population genetics can inform an understanding of the drivers of dispersal and dynamics of population growth, and the relative importance of such factors in a system with multiple immigration pathways, differing levels of multidirectional movement and extinction/recolonization dynamics all placed within a highly fragmented landscape. By definition, invasive pest species exhibit characteristics such as high levels of propagule pressure promoted by the use of multiple dispersal pathways and genetic and life-history characteristics that favour establishment success and persistence (Lockwood et al. 2007). Therefore, it becomes important to incorporate information as we have into an invasive species management strategy to ensure that sufficient effort is placed where required, thus maximizing the likelihood for successful control outcomes.

## Data archiving statement

Data for this study are available at the Tropical Data Hub (TDH), which is located at: https://eresearch.jcu.edu.au/tdh//.
